# When is it worth being a self-compatible hermaphrodite? Context-dependent effects of self-pollination on female advantage in gynodioecious *Silene nutans*

**DOI:** 10.1002/ece3.1410

**Published:** 2015-04-11

**Authors:** Emna Lahiani, Pascal Touzet, Emmanuelle Billard, Mathilde Dufay

**Affiliations:** Unité Evolution Ecologie Paléontologie, UMR CNRS 8198, Université de Lille 1 - Sciences et TechnologiesVilleneuve d’Ascq, France

**Keywords:** Female advantage, gynodioecy, male sterility, pollen limitation, self-pollination, *Silene nutans*

## Abstract

In gynodioecious plant species with nuclear-cytoplasmic sex determination, females and hermaphrodites plants can coexist whenever female have higher seed fitness than hermaphrodites. Although the effect of self fertilization on seed fitness in hermaphrodites has been considered theoretically, this effect is far from intuitive, because it can either increase the relative seed fitness of the females (if it leads hermaphrodites to produce inbred, low quality offspring) or decrease it (if it provides reproductive assurance to hermaphrodites). Hence, empirical investigation is needed to document whether relative seed fitness varies with whether pollen is or is not limiting to seed production. In the current study, we measured fruit set and seed production in both females and hermaphrodites and the selfing rate in hermaphrodites in two experimental patches that differed in sex ratios in the gynodioecious plant *Silene nutans*. We found an impact of plant gender, patch, and their interaction, with females suffering from stronger pollen limitation when locally frequent. In the most pollen-limited situation, the selfing rate of hermaphrodites increased and provided hermaphrodites with a type of reproductive assurance that is not available to females. By integrating both the beneficial (reproductive assurance) and costly effects (through inbreeding depression) of self-pollination, we showed that whether females did or did not exhibit higher seed fitness depended on the degree of pollen limitation on seed production.

## Introduction

The evolution from hermaphroditism towards separate sexes is considered a major evolutionary transition in the history of flowering plants (Barrett 2010). One possible evolutionary step within that transition is the evolution of gynodioecy, that is the co-occurrence of female and hermaphroditic individuals within the same population (Charlesworth and Charlesworth 1978). This evolutionary pathway does not seem to be independent from a plants’ ability to self-pollinate, which is another key characteristics of plant reproduction. Indeed, most of the well-documented gynodioecious species are self-compatible, while gynodioecious species in which hermaphrodites are unable to self-pollinate appear very rare (Dufay and Billard 2012), suggesting that separate sexes may evolve more easily in self-compatible lineages. Accordingly, both theoretical (e.g. Charlesworth and Charlesworth 1978) and empirical studies (reviewed in Dufay and Billard 2012) have shown that the maintenance of females within a hermaphroditic population should be facilitated if hermaphrodites partly self-pollinate, because hermaphroditic progeny may therefore suffer from inbreeding depression. Such inbreeding avoidance is one way for females to benefit from a so-called “female advantage”, and thus compensate for their fitness loss in terms of pollen production. However, in case of pollen limitation, the ability to self-pollinate may provide hermaphrodites with a reproductive assurance, therefore reducing the magnitude of the female advantage. Although the positive effect of selfing on reproductive assurance for hermaphrodites of gynodioecious species has been hypothesized by several theoretical studies (Charlesworth, 1981; Maurice and Fleming 1995; Dornier and Dufay 2013), empirical investigation of such an effect is lacking. Furthermore, because self-pollination potentially can both increase the relative fitness of females versus hermaphrodites (in case of inbreeding depression) and decrease it (in case of pollen limitation), whether or not gynodioecy is more likely to be maintained in a self-compatible lineage is clearly not intuitive.

The aim of this study is to empirically disentangle the positive and the negative effects of self-pollination in hermaphrodites on the female advantage in a gynodioecious species. In particular, we aimed at testing whether self-pollination provides hermaphrodites with a reproductive assurance in pollen-limited situations. We thus measured seed production in females and hermaphrodites as well as the selfing rate in hermaphrodites when pollen limitation varies. We carried out the study in two patches exhibiting different sex ratios, since a low proportion of hermaphrodites in a local context is expected to enhance pollen limitation in gynodioecious species (e.g. Graff 1999; Alonso 2005; Zhang et al. 2008; De Cauwer et al. 2010). Our study was carried out in semi-controlled conditions, in order to control for other parameters that might affect seed fitness, such as habitat quality or pollinator availability, and to allow the two experimental populations to differ only in the magnitude of pollen limitation. We then combine our empirical measurements of seed production, self-pollination and a previous estimation of inbreeding depression to estimate the magnitude of the female advantage.

## Materials and Methods

### Study species

Our study was performed using the gynodioecious, entomophilous and self-compatible *Silene nutans* (Caryophyllaceae). *Silene nutans* (Caryophyllaceae) is a diploid, long-lived perennial rosette plant growing in dry, open-grass communities of hillsides. It is described as gynomonoecious-gynodioecious, with female, gynomonoecious (plants bearing both perfect and pistillate flowers), and hermaphroditic individuals found in natural populations (Jurgens et al. 2002; Dufay et al. 2010). The determination of sex is nuclear-cytoplasmic, with cytoplasmic sterility factors that can be counteracted by nuclear restorers (Garraud et al. 2011), although the determination of the gynomonoecious phenotype is not known yet. With such genetic determination, theory has shown that the maintenance of females only requires a slightly higher seed fitness in females compared to hermaphrodites (Lewis 1941; Gouyon et al. 1991). Sex ratio varies among populations from 0 to 60% of female plants and from 0 to 100% at the level of patches of plants (Dufay M, unpubl. data). Population size is highly variable and can be extremely small (Hauser and Weidema 2000; Van Rossum and Prentice 2004). Flowers are visited by a number of different insect species, including Noctuidae, Sphingidae, Hymenoptera, and nectar robbing Hymenoptera (Jurgens et al. 1996). Hermaphrodites produce larger flowers than females (Dufay et al. 2010) but the number of ovules per flower does not vary between sexes (Dufay M, unpubl. data). Perfect flowers are protandrous, which makes autonomous selfing unlikeley, but self-pollination can occur by geitonogamy.

### Plant material

Individual plants used in the experiment were sampled in 2008 from natural populations. All plants were cloned from plantlets grown and over-wintered in the greenhouse for 10 weeks during the winter of 2010. Plants were then potted in a soil mix (3/4 compost; 1/4 perlite) and placed in greenhouse at a temperature of 20°C for 7 weeks until the population reached the peak of flowering. All focal plants used in this study originated from a single wild population in Olloy-sur-Viroin in Belgium (Van Rossum et al. 1997), which contained *ca* 400 individuals and a female ratio of 0.16 during the survey in 2008, with local female frequency varying from 0 to *ca* 0.7 (Dufay M, unpubl. data). To properly establish our experimental populations in terms of number of individuals and sex ratio (see below), we also used eight genotypes from our collection, originating from other localities in Germany, France and United Kingdom (Table S1); these were assigned randomly to each experimental patch.

### DNA extraction and genotyping

All hermaphroditic plants (i.e. potential fathers of seeds produced in each experimental population, see below) were genotyped in order to estimate the magnitude of self-pollination. For this purpose, DNA was extracted from 100 to 150 mg of leaf tissue using Macherey-Nagel Nucleospin, Düren,Germany 96 Plant II Kits. A DNA sample from each plant was assayed for five scored microsatellite loci (for primers see Table S2). Amplification reactions were carried out by polymerase chain reaction (PCR) with 20 ng of DNA in 10 *μ*L reaction volume containing 5 *μ*L of Qiagen multiplex Kit 2x, 1 *μ*L of primer mix (10x) (0.75 *μ*mol/L of Scored forward primer and 3.75 *μ*mol/L of reverse primer) and 1 *μ*L of sterile water. Cycling conditions for PCR amplification were 95°C for 15 min, five cycles of 45 sec at 95°C, 5 min at 68°C with a step-down of 2°C per cycle, 1 min at 72°C, fives cycles of 45 sec at 95°C, 5 min at 58°C with a step-down of 2°C per cycle, 1 min at 72°C, 27 cycles of 45 sec at 95°C, 30 sec at 47°C, 1 min at 72°C and finally 72°C for 10 min. Amplification products were separated on Applied Biosystems 3130 capillary sequencer. Raw data were analysed using GeneMapper version 3.5 (Applied Biosystems, Applied Biosystems, Villebon sur Yvette, France). Individuals with doubtful or missing peaks or for which mismatches occurred between mothers and progeny were genotyped a second time.

### Experimental populations

We set up our experiment in a common garden located on the campus of the University of Lille, France. This site contains several green areas, and both nocturnal and diurnal insect visitors were observed on patches of *Silene nutans*. We created two experimental patches, in two sites of the garden that were equally distant from buildings and green areas and were separated by *ca* 40 meters in order to create two relatively independent pollination contexts. A total of 144 potted plants (72 females and 72 hermaphrodites) were used in the experiment. Both patches contained 72 plants, exhibited the same plant density and a similar number of open flowers (see below), but they were designed to exhibit different local sex ratios, the first one being female-biased (hereafter FB patch, with 85% of individual plants - 60 females out of 72 plants - and 78% of flowers being female), while the second one being hermaphrodite-biased (hereafter HB patch, with 85% of individual plants - 60 hermaphrodites out of 72 plants - and 87% of flowers being hermaphroditic). In each of the two patches, 36 individual plants were followed. These focal plants were placed in the centre of each patch and were rotated every 2 days, by randomly assigning to each plant one of the 36 locations in the center of the patch. The other 36 individual plants were randomly placed around the focal individuals. All plants were watered every day.

To construct the two experimental patches, individual plants were chosen according to several criteria. First, we aimed to exclude gynomonoecious plants, in which the proportion of female flowers varies from 0.03 to 0.9 (Dufay et al. 2010) and also varies through time, which could modify local sex ratios. An extensive phenotyping of plant gender in our collection, performed during the previous flowering season, allowed us to retain only pure females and pure hermaphrodites for this experiment. Second, we retained only plants that were at their peak of flowering at the beginning of the experiment and made sure that the overall number of flowers was similar between the two patches (2350 and 2570 open flowers in the FB and the HB patches, respectively).

### Pollen limitation and pollination success

From 20th May 2010, 72 focal individual plants (12 hermaphrodites and 24 females in the FB patch; 12 females and 24 hermaphrodites in the HB patch) were placed for open pollination for one entire week. On the first day of the experiment, three flowers were marked at bud stage on each focal plant. These flowers were left untreated, thus receiving only natural levels of pollination (hereafter open-pollinated flowers). On a subsample of the focal plants (12 hermaphroditic plants and 12 female plants in each patch), we additionally marked three other flowers at bud stage. These flowers were hand-pollinated once a day, by gently brushing receptive stigmas with fresh ripe pollen collected every day from 13 hermaphrodites, which were growing in a greenhouse and were different from the plants located in the two experimental patchs. We thus had two categories of focal plants: manipulated plants bearing three open-pollinated and three hand-pollinated flowers (12 hermaphroditic plants and 12 female plants in both patchs) and control plants on which only three open-pollinated flowers were followed (12 female plants in the FB patch and 12 hermaphroditic plants in the HB patch). On the two types of marked flowers (open-pollinated and hand-pollinated), we estimated the fruit set as the proportion of marked flowers setting fruit 2 weeks later. At that time, to prevent seed loss, fruits were covered with mesh bags before they opened. Fruits were then collected as they matured, 4 weeks after pollination; seeds were counted and the weight of all seeds per fruit was recorded. For each focal plant and for each treatment, we thus obtained one average value of seed number per fruit, and one average value for the weight of one seed. We estimated the pollination success as the product: fruit set × average number of seeds per fruit. This was calculated for each treatment (open/hand-pollinated), for each plant.

Plants are considered as pollen limited if additional pollen increases fruit or seed production (Schemske 1980; Willson and Schemske 1980; Bierzychudek 1981; Burd 1994; Larson and Barrett 2000). We thus analyzed the occurrence of pollen limitation by comparing open-pollinated and hand-pollinated flowers within each manipulated plant for their fruit set, their average seed number and their pollination success, by performing paired *t*-tests. We performed four different analyses, one per plant gender and per patch. One must note, however, that when additional pollen is applied to only some flowers on a plant, resources may be shunted away from untreated flowers. The immediate increase in seed production due to supplemental pollination as a measure of pollen limitation is thus potentially confounded with this compensation among flowers (Ashman et al. 2004). To assess this possible mechanism, we compared open-pollinated flowers of control plants and open-pollinated flowers of manipulated plants for their fruit set and their seed production, in each patch and for each plant gender. If there was some compensation, one should record a higher seed production in open-pollinated flowers of control plants compared to open-pollinated flowers of manipulated plants. Finally, we analyzed the effect of patch, plant gender, and their interaction on fruit set, average seed number per fruit and resulting pollination success for all surveyed open-pollinated flowers. Analyses of fruit set were performed by using a logistic regression (binomial distribution, log link function, proc GENMOD, SAS, Cary, NC), correcting for overdispersion (dscale option, proc GENMOD, SAS). Average seed weight, average seed number per fruit and pollination success were performed by using an ANOVA (proc GLM, SAS). In order to reach a normal distribution of residuals, we log-transformed the average seed number and the pollination success (Kolmogorov-Smirnov test of normality: *P *>* *0.15 for all shown analyses). All statistical analyses were conducted using SAS (SAS version 9.1.3, 2002).

### Estimation of selfing rates

We estimated the selfing rate of the seeds produced by open-pollinated flowers in 35 out of the 36 focal hermaphrodites (23 individuals in the *HB* patch and 12 individuals in the *FB* patch). From 15 to 50 seeds per plant depending on seed production were sown in Petri dishes on Whatman paper. Filter papers were kept moist during germination. The location of the dishes was regularly changed. After 12 days, up to 50 seedlings from each plant were randomly selected, transplanted into a soil mix (3/4 compost; 1/4 perlite), and placed at 20°C with daily moistening to minimize any stress of transplantation. Six weeks later, plantlets were collected in order to extract their DNA by using MACHEREY-NAGEL NucleoSpin® 96 Plant II Kits. 1012 plantlets from the 35 families were genotyped by the five scored microsatellites loci described previously. Outcrossing rates were determined using Ritland (2002) multilocus maximum likelihood estimation program (MLTR Version 3.4, accessible at http://genetics.forestry.ubc.ca/ritland/programs.html). Standard deviations were determined based on 1000 bootstrap analyses; maternal genotypes were estimated as part of the maximum likelihood procedure. Within each patch, family estimates gave outcrossing rates per family, bootstraps using individual offspring as units of observation. We calculated selfing rate as *s *=* *1 − *t*_m_.

Differences in selfing rate between the two patches were analyzed by using a logistic regression (binomial distribution, log link function, proc GENMOD, SAS), correcting for overdispersion (dscale option, proc GENMOD, SAS). Moreover, we used these results to estimate the number of seeds produced by outcrossing in each focal plant. To do so, we multiplied the average seed number per fruit by the outcrossing rate for each hermaphroditic plant. We performed the same calculation for female plants, by considering the outcrossing rate to be 1. On these new data, we tested the effect of patch, plant gender and their interaction by using proc GLM (SAS), as explained previously. Log transformation did not help in reaching normal distribution of residuals in that case. We thus present the analyses for non transformed data, but with a non-normal distribution of residuals (Kolmogorov-Smirnov, *P *<* *0.01).

## Results

Fruit set of open-pollinated flowers did not significantly differ between control and manipulated plants in the FB patch (12 manipulated females vs. 12 control females: 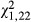
* *= 1.98, *P *=* *0.159) or in the HB patch (12 manipulated hermaphrodites vs. 12 control hermaphrodites: 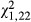
* *= 1.39, *P *=* *0.238). The same result was found for the average seed number per fruit (FB patch: *F*_1,22_ = 3.39, *P *=* *0.079; HB patch: *F*_1,22_ = 1.69, *P *=* *0.206). It is thus unlikely that open-pollinated flowers to which hand-pollinated flowers were compared within manipulated plants suffered from lower resources allocation. This suggests that any difference between these two types of flowers can be attributed to pollen limitation. Focusing on manipulated plants, we found a significantly larger fruit set in hand-pollinated flowers compared to open-pollinated flowers carried by the same plant, for focal female plants in both patches and for focal hermaphrodites in the FB patch only, indicating some pollen limitation affecting fruit set in these three categories of plants (Table1). Evidence for pollen limitation was also found on the number of seeds per fruit and on the pollination success (fruit set × seed number) for both plant genders in both patches (Table1). However, seed number per fruit was extremely variable among hand-pollinated flowers (from six seeds to 128 seeds per fruit), even for a given plant gender in one particular patch, which could be due to potential failure in hand pollination. Thus, the magnitude of pollen limitation may have been underestimated and the difference between the number of seeds produced by hand-pollinated and open-pollinated flowers could not be analyzed further, as it was not a reliable estimation of the intensity of pollen limitation. Thus, to compare pollination efficiency among genders and patches, we then focused on open-pollinated flowers (that were on average limited in pollen supply, according to our results).

**Table 1:** Results of paired *t*-tests comparing pollination efficiency between hand-pollinated and open-pollinated flowers within manipulated plants. *D* is the difference for all variables between the two pollination treatments (hand pollinated – open pollinated) and the test indicates whether this difference is significantly different from 0. For each variable, four analyses were run, one per plant gender and experimental population

Variable	*D*	*t*	*P*
Fruit set			
Female plants in the FB patch	0.21	2.90	0.014
Hermaphroditic plants in the FB patch	0.20	2.52	0.02
Female plants in the HB patch	0.11	2.33	0.04
Hermaphroditic plants in the HB patch	0.01	0.16	0.87
Average seed number per fruit			
Female plants in the FB patch	58.25	5.74	0.0001
Hermaphroditic plants in the FB patch	51.26	8.41	<10^−4^
Female plants in the HB patch	54.55	8.41	<10^−4^
Hermaphroditic plants in the HB patch	56.97	8.20	<10^−4^
Pollination success (fruit set × average seed number)			
Female plants in the FB patch	55.44	5.90	0.0001
Hermaphroditic plants in the FB patch	52.98	7.04	<10^−4^
Female plants in the HB patch	55.04	8.45	<10^−4^
Hermaphroditic plants in the HB patch	49.05	9.08	<10^−4^

**Figure 1:**
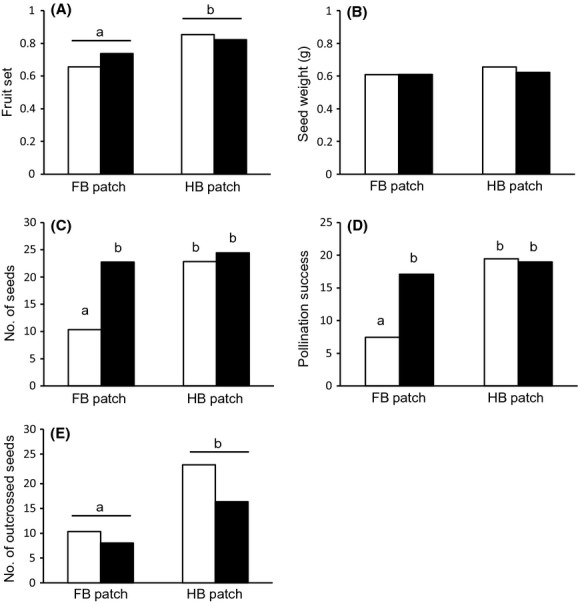
(A) fruit set as the proportion of marked flowers setting fruit 2 weeks later, (B) average weight of one seed per fruit, (C) average number of seeds per fruit, (D) pollination success (fruit set × average seed number) and (E) average number of outcrossed seeds per fruit, of open-pollinated flowers, as a function of plant gender and patch (FB: female biased, HB: Hermaphrodite biased). White: female plants, black: hermaphroditic plants. Letters indicate significant differences among levels, according to the post-hoc Tuckey tests.

Regarding open-pollinated flowers, fruit set was significantly lower in the FB patch compared to the HB patch (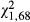
* *= 5.80, *P *=* *0.016) but was not affected by plant gender (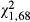 = 0.04, *P *>* *0.1) nor by the interaction between the two factors (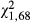 = 0.77, *P *>* *0.1). Average seed number and pollination success were both significantly lower in female plants and in the FB patch (Table2). These two variables were also affected by the interaction between the two factors (Table2), with post-hoc pairwise comparisons showing significantly lower values on female plants in the FB patch compared to the other three plant categories (Fig.1). Finally, the average weight of one seed did not significantly differ by patch or plant gender (Table2), suggesting that resource reallocation did not increase seed quality in plants experiencing stronger pollen limitation.

**Table 2:** Results of the analyses of variance on the various estimates of pollination efficiency on open-pollinated flowers

Variable and sources of variation	df	MS	*F*	*P*
Average seed number per fruit^1^				
Plant gender	1	0.799	9.95	0.0024
Patch	1	0.563	7.02	0.01
Plant gender × patch	1	0.485	6.05	0.0165
Error	68	0.080		
Average seed number produced by outcrossing				
Plant gender	1	296.70	2.89	0.09
Patch	1	1744.88	17.00	0.0001
Plant gender × patch	1	63.57	0.61	0.43
Error	67	102.62		
Average weight of one seed				
Plant gender	1	0.06	1.44	0.23
Patch	1	0.09	2.08	0.15
Plant gender × patch	1	0.07	1.5	0.22
Error	68	0.04		
Pollination success (fruit set × average seed number)^1^				
Plant gender	1	0.948	8.44	0.005
Patch	1	1.259	11.21	0.0013
Plant gender × patch	1	0.747	6.65	0.0121
Error	68	0.112		

1 log transformed.

The selfing rate of hermaphroditic plants could be estimated on 12 families produced by focal hermaphroditic plants in FB patch and on 23 families in the HB patch. We found that hermaphrodites experiencing a high local female frequency self-pollinated at a significantly higher rate that the other group (*s *=* *0.59 ± 0.34, min = 0, max = 1 in the FB patch; *s *=* *0.28 ± 0.26, min = 0; max = 0.9 in the HB patch; 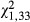 = 10.82, *P *=* *0.0045, see also Table3). Finally, the number of outcrossed seeds (estimated on the basis of the selfing rate in each family) was significantly higher in the *HB* compared to the *FB* patch, but it was only marginally affected by plant gender and it did not depend on the interaction between the two factors (Table2, Fig.1).

**Table 3:** Multilocus (*t*_m_) and mean single locus (*t*_s_) outcrossing rates and biparental inbreeding (*t*_m_ *− t*_s_) estimated in the two populations (HB: hermaphroditic-biased; FB: female-biased)

	HB patch	FB patch
Families (progenies)	23 (712)	12 (300)
*t*_m_ (SD)	0.653 (0.070)	0.492 (0.071)
*t*_s_ (SD)	0.477 (0.053)	0.446 (0.079)
*t*_m_ *− t*_s_ (SD)	0.176 (0.025)	0.046 (0.019)
Family *t*_m_ range (*N*)	0.041–1 (23)	0–1 (12)

## Discussion

The aim of this study was to investigate whether females benefit from a seed fitness advantage relatively to self-compatible hermaphrodites, and how self-pollination impacts this parameter. We thus measured how seed production and self-pollination varied between two experimental patches, characterized by the same size and density, the same soil quality but with very different sex ratios. For practical reasons, we decided to exclude gynomonoecious individuals from this experiment. It is however likely that high frequencies of gynomonoecious plants (as it has been observed in some populations, Dufay M, unpubl. data) could affect both pollen limitation and average selfing rate at the population level (e.g. Collin and Shykoff 2003). The global impact of gynomonoecious individuals on the dynamics of male sterility should thus be investigated in further studies. The aim of the study was not to directly investigate the impact of sex ratio on seed production; we used different sex ratios in order to create different situations in terms of pollen limitation. Our experimental patches were of a moderate size, with relatively high plant and flower density, thus mimicking some of the very diverse situations that have been observed in natural populations of *Silene nutans*, or patches within these populations (Dufay M, unpubl. data). According to our results, the plants located in our experimental patches experienced some pollen limitation, which explains the overall low values of fruit set and seed number. Pollen limitation has been documented in several species (reviewed in Knight et al. 2005), including gynodioecious ones (e.g. Graff 1999; Alonso 2005; De Cauwer et al. 2010) and it thus very likely that our experiment reflects some natural situations in *Silene nutans*, in particular for populations of the species that show small size and/or some degree of isolation (Hauser and Weidema 2000; Van Rossum and Prentice 2004). We found that the intensity of pollen limitation varied according to the experimental patch, plant gender and their interaction. In all cases, the average seed weight did not differ between genders or between experimental populations, suggesting that plants experiencing the highest level of pollen limitation (i.e. females in the female-biased population, see below) did not increase the quantity of resources within seeds produced. Thus, the differences in fruit set and seed number between genders are likely to represent an actual difference in offspring production.

An effect of plant gender was detected, with female plants exhibiting overall lower seed number per fruit and lower pollination success compared to hermaphrodites. This result could be a consequence of the smaller flower size in female individuals, which has been reported in this species (Dufay et al. 2010) as in many other gynodioecious species (reviewed in Shykoff et al. 2003). Pollinators might be less attracted to the small flowers, which may also produce less nectar or fewer attractive volatile compounds (as found, for instance, in dioecious *Silene latifolia*: Waelti et al. 2009). Pollinator preference for hermaphroditic flowers is a common result in gynodioecious species (e.g. Delph and Lively 1992; Williams et al. 2000; Asikainen and Mutikainen 2005; Griffin and Byers 2012) and it sometimes translates into more pollen limitation for female individuals (e.g. Alonso 2005 but see Shykoff et al. 2003). A difference between the two experimental patches was also detected on all pollination measurements (i.e. fruit set, seed number and resulting pollination success). Although we cannot reject that other non-controlled differences between patches led to such variation in pollination quality, this result might suggest an effect of the local sex ratio on the intensity of pollen limitation, which could be explained by an overall lower attractivity of the female-biased population and/or by a lower local availability of pollen in this population. Such effect of sex ratio on pollen limitation has been predicted by several theoretical studies (e.g. Lewis 1941; Maurice and Fleming 1995; McCauley and Taylor 1997) and empirically investigated, whereas not always found, in several gynodioecious species (e.g. Cuevas et al. 2008; Case and Ashman 2009; see also the reviews by Shykoff et al. 2003 and Ashman 2006).

Whatever the proximal causes for this difference in pollination between the two patches, the strong interaction between patch and plant gender that was detected on seed number per fruit and on pollination success shows that pollen limitation affects the two genders differently. A few other studies have reported similar results - Widen and Widen (1990) in *Glechoma hederacea*, McCauley and Brock (1998) in *Silene vulgaris* and Zhang et al. (2008) in *Glechoma longituba* - and sometimes postulated that this pattern may be explained by the ability to self-pollinate, which would provide hermaphrodites with a reproductive assurance in pollen-limited situations. With the current study, we had the opportunity to directly test this hypothesis. We found that hermaphrodites do self-pollinate, although selfing rates were extremely variable among plants. Because flowers are protandrous, most of the self-pollination events are likely to be performed through geitonogamy. The observed variability in selfing rates in this study could thus reflect some stochasticity related with pollinator movements. We found that hermaphroditic plants had different selfing rates between the two patches, which can either be explained by the difference in sex ratio or by some uncontrolled difference between the two situations. Noteworthy, in gynodioecious *Silene vulgaris* Miyake and Olson (2009) found an effect of population sex ratio on selfing rate in hermaphrodites (see also the review by Ashman 2006). In both their study and the current study, such result can be explained by a higher probability of receiving self-pollen through geitonogamous pollination when hermaphrodites are rare. In the current study, the novelty was that we could directly show that such frequent self-pollination explains why hermaphrodites suffer less from pollen limitation. When focusing on seeds produced by outcross pollination (thus, artificially removing the effect of self-pollination in hermaphrodites), we found only a marginal effect of plant gender and no effect of the interaction. In other words, if one removes seeds produced by self-pollination, hermaphrodites lose their advantage in terms of seed production, in particular in the female-biased population that exhibits strong pollen-limitation. To our knowledge, this is the first direct evidence of self-pollination providing hermaphrodites with a reproductive assurance in a gynodioecious species.

One remaining question is whether the ability to self-pollinate is an overall benefit or a detriment for hermaphrodites. Indeed, even though self-pollination increases seed production and apparently provides hermaphrodites with a fitness advantage compared to females, inbreeding depression may decrease offspring quality and lead to the opposite effect. The magnitude of the female advantage will therefore depend on the balance of these two effects of self-pollination, as shown by Equation 1,

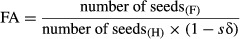
1

with *s* being the selfing rate of hermaphrodites and *δ* the magnitude of inbreeding depression. Following Equation 1, we calculated the magnitude of *FA* in the two patches, by using the values of selfing rate obtained in the current study, as well as the values of pollination success as an estimate for the number of seeds produced by females (F) and hermaphrodites (H). When considering all possible values of *δ* between 0 and 0.9, we found that *FA* varied from 0.43 to 0.93 in the FB patch, and between 1.05 and 1.41 in the HB patch. In particular, when considering *δ *= 0.3, following Dufay et al. (2010), who investigated inbreeding depression in *S. nutans*, we found a female advantage of 0.53 and of 1.15 in the female-biased patch and in the hermaphroditic-biased patch, respectively. This suggests that in conditions mimicked by the hermaphroditic-biased patch, females benefit from a female advantage >1, which is a sufficient condition for a cytoplasmic gene of male sterility to invade and be maintained (Lewis 1941). Hence, although hermaphrodites produce more seeds, inbreeding depression may be strong enough to outweigh this fecundity advantage. Females may be maintained or even spread, therefore, despite producing fewer seeds, if selfed hermaphrodite seeds suffer strong enough inbreeding depression. In contrast, in the female-biased patch, pollen limitation experienced by females may be too strong, such that higher offspring quality would not be sufficient to provide them with higher relative seed fitness compared to hermaphrodites. If this were the case, the ability to self-pollinate would benefit hermaphrodites. Our results thus show that in some situations self-pollination provides hermaphrodites with a reproductive assurance that is not available to females, while the ability of selfing may be globally detrimental for hermaphrodites in others environments. Although this remains to be directly tested, this lends credence to the hypothesis that the capacity of a male-sterility mutation to invade a population of self-compatible hermaphrodites will vary according to the pollination environment, and possibly, to the local sex ratio.

## Supporting Information

**Table S1.** List of populations from which individual plants were collected. **Table S2.** Primer list of microsatellites loci.
