# Supplementary material for: When is it worth being a self-compatible hermaphrodite? Context-dependent effects of self-pollination on female advantage in gynodioecious Silene nutans
Source: Ecol Evol. 2015 Apr 11;5(9):1854–62. doi: 10.1002/ece3.1410 (PMC4485966; doi:10.1002/ece3.1410)
Supplement: Supplementary file 1 [file ece30005-1854-sd1.docx]

**Supplementary Table 1**: **Origin of plant material used in the study.**

| **Patch** | **Plant types** | **Localization (N)** |
| --- | --- | --- |
| Female biased | Female focal plants | Olloy, Belgium (24) |
|  | Hermaphrodite focal plants | Olloy, Belgium (12) |
|  | Female non focal plants | Olloy, Belgium (32)  Devèze, France (1)  Aussois, France (1)  Littlehampton, UK (1)  Swanage, UK (1) |
| Hermaphrodite biased | Female focal plants | Olloy, Belgium (12) |
|  | Hermaphrodite focal plants | Olloy, Belgium (24) |
|  | Hermaphrodite non focal plants | Olloy, Belgium (32)  Vireux, France (1)  Loffenau, Germany (1)  Champtoceaux, France (1)  Moëlan sur mer, France (1) |

**Supplementary table 2. Primer list of nuclear microsatellite loci**

| **Loci** | **Forward sequence** | **Reverse sequence** |
| --- | --- | --- |
| **B09** | AAGGGCACAAAATTGAGAAGG | GTGTCTTCCAAAGGTGAAGCTCATATAAACC |
| **E08** | GTTGGTCGTTGGTAGTTCACAG | GTGTCTTAATGCGAATCGGTCAATTTTAC |
| **G01** | CCCTACCTCATAGCAACAAGC | GTGTCTTCCTTCTCCTCCTTCCTTTAACC |
| **H07** | AAGCAAAACCCCTTATAAGCATC | GTGTCTTACCTTTCCCCTTCCTCCTTT |
| **D10** | CGGGCTAAGTTTACAGCATCA | GTGTCTTTGCCGTTATGCCATTCATTA |
